# A Course of Clinical Lectures, Delivered at the Hôtel Dieu, Paris, for the Session 1842-’43

**Published:** 1843-05-13

**Authors:** A. F. Chomel

**Affiliations:** Paris


// THE MEDICAL EXAMINER,

And Retrospect of the Medical Sciences.

Vol. VI.] PHILADELPHIA, SATURDAY, MAY 13, 1843. [No. 9.

### A COURSE OF CLINICAL LECTURES,

*Delivered at the Hôtel Dieu, Paris, for the Session* 1842–'43.

BY A. F. CHOMEL, M. D.

### LECTURE VII.

#### HYSTERIA—RAMOLLISSEMENT.

At No. 4 of the Salle Saint-Bernard you will find a young girl, of strong constitution, who was brought to the hospital about eight days since in nearly a comatose state. She was suffering from a nervous attack which had just seized her, and the characters of which we will now proceed to examine, as some interesting circumstances were connected with it, which complicated it, and rendered the diagnosis by no means easy.

On her entrance into the hospital she was insensible, or rather she appeared unconscious of what was going on around her, without being, at the same time, in a state of actual coma. Finding that there was no immediate danger, and that the affection was only nervous, probably hysteria, we abandoned the patient to the unaided efforts of nature, limiting ourselves to recommend to those in charge of the service the necessary watch to prevent accident. However, on seeing such attacks in a young girl, in such apparent robust health, we subsequently began to suspect that there might be something more than simple hysteria; to satisfy ourselves on this point, we availed ourselves of a moment of remission and calm to question the patient upon some points of her antecedent history. She gave us in answer the following details.

She is a mantua-maker by trade, and has inhabited Paris for a short time only, living before in the country. Her present affection first appeared at sixteen years of age, and was attributed by her to the following circumstance: One day, whilst in the fields, at some distance from home, she was attacked by a dog, who did her no bodily harm, but frightened her excessively. As soon as she returned home she immediately experienced in her whole body irregular, involuntary movements: in fact, genuine convulsions. The termination of this first attack was followed by an acute affection, with nervous symptoms, which yielded to appropriate treatment. These nervous symptoms were renewed for two years at irregular intervals, but always without fever. At nineteen years of age she had, for the first time, her catamenia; and in spite of the establishment of this new function, the nervous phenomena continued, and appeared even to augment. These periodical attacks finally exposed her, as so often happens in small places, to isolation, and to the reproaches of her neighbours, which finally induced her to quit her native spot and settle in Paris. Arriving here, she soon entered a shop as an attendant, but her nervous attacks again recurring, and so violently and constantly, that she determined on quitting her employment and becoming a seamstress—an occupation which enabled her to take better care of herself, and to hide her attacks from the public eye. Nevertheless the attacks continued, their frequency and intensity increasing. We have asked her if she was attacked indiscriminately, wherever she might be, in spite of herself, and without her being able to oppose them. She answered that she was constantly attacked out of doors, in public, without its being in her power in any manner to prevent them; she told us that during the attacks she saw and heard all that passed around her, but without being able to indicate her consciousness.

At the commencement of the attack she experiences a violent pain in the epigastric region, which gradually ascends towards the throat, impeding respiration; and she then falls without apparent consciousness. At other times a spasm seizes the throat, suffocating her, and is followed by convulsions. Since the invasion of these attacks her disposition has materially changed; she has become very impressionable; the slightest opposition excites her, and produces an attack. Menstruation does not seem to exercise any marked influence upon the production of these accidents, for they frequently occur at the menstrual period and continue throughout. After an attack has taken place, her mouth ordinarily remains a little drawn to the left side; there is some degree of hesitation in the speech; at the same time there is some *gêne* in motion, with a little obtuseness in sensibility. This diminution in sensibility and motion is more marked in the left side of the body than the right. It seems to me also that she sustains herself less well on her left leg than on her right. She sometimes has violent cephalalgia, principally at the top of the head.

All these symptoms induce us to believe in an hysterical affection, but complicated with an epileptiform tendency. These attacks seize her suddenly, in the midst of the street, and wherever she may be, without her being able in any manner to prevent them, or to moderate their intensity. This circumstance militates strongly in favour of the epileptiform character of the complaint, and hence results the suspicion of an organic affection of the brain, which probably is its cause. It is almost unexampled for an hysterical woman to have a paroxysm in the street, and in places where she may be exposed to the public gaze. Hysterical paroxysms can always be voluntarily adjourned, or at least for a sufficient length of time to permit those who are attacked by them to retire into privacy; whilst attacks of epilepsy are instantaneous, and entirely involuntary.

The attacks, then, from which this patient suffers, are of the nature of epilepsy, and consequently, we should admit in her the probability of a cephalic lesion—for they are almost invariably due to the presence of a tumor in the brain, or of a cyst compressing some portion of the nervous matter.

Much is to be apprehended from the sequelæ of these attacks. This kind of complication of hysteria and epilepsy is by no means assuring.

It has been said that sexual appetite is one of the determining causes of hysteria, and that it most frequently occurs in women who are deprived of sexual indulgence. Hence, the cure has been found in the union of the sexes. Now I regard this view as altogether erroneous. You constantly see attacks of hysteria in married women, and they are not rare in prostitutes, and in women who commit more or less venereal excess.

On the other hand, you constantly meet with hysteria in women who are a prey to some mental suffering; in young women who are married against their will, or in girls who have been disappointed. From these circumstances one is led to regard hysteria more frequently as rather the result of moral influences than physical causes. Finally, some women fall into hysteria at the moment of coition, so that in these you are unable to produce at all venereal excitement, without a paroxysm.

I regard, then, hysteria, in a word, as a disease essentially nervous, altogether independent of uterine influence, and which may occur as well in the male as the female, if the impressionability of the nervous system of the two sexes were the same. But the different temperament of man, the greater sturdiness of his character, and his inferior excitability, predispose him rather to hypochondria, which in him seems to be analogous to hysteria in the female.

As to our patient, you see manifestly that in her the hysteria was produced by a moral cause, by violent fright, which gave a great shock to her nervous system. We questioned her with regard to the fact of her ever having had sexual relations, being induced to do this from the fact which I mentioned above,—from the popular prejudice which many physicians follow, which regards them as salutary in this class of affections. She answered in the affirmative, adding that it had been by the advice of her physician. I do not here wish to enter into any discussion regarding the question of morality, although this is a question of great gravity, but will content myself with saying that such advice is absurd, and as prejudicial in a physical point of view as in a moral. How can you foresee to what this advice may lead; and besides, how many women are there to whom sexual intercourse is positively injurious! No physician, who respects himself, should ever attempt to give such advice.

At No. 1 of the Salle Saint-Agnes you have remarked a strong, robust man, of about forty years of age, suffering from a grave affection. Twenty years ago he had a severe attack of articular rheumatism in his left elbow, which yielded, however, to appropriate treatment, without leaving any apparent trace of disease of the heart. For the last ten years he has habitually suffered from severe pain in the head, the intensity of which, however, varied. There is sometimes deviation of the mouth to the left side, at the same time that his left arm becomes more feeble than his right. These symptoms, which were in a measure remittent, augmented in intensity within the last four years, during which time he has remained in a constant state of dizziness, with general feebleness, more particularly of the left side of the body, which have prevented him from attending to his occupations. About two years since he had a fall on his side, which obliged him to remain in bed for two weeks, at the end of which time he believed himself entirely recovered; but soon afterwards his former symptoms returned, and made slow but sensible progress.

On the 20th of last September he had abundant diarrhœa, with vomiting; the cephalalgia and other cerebral symptoms continuing to manifest themselves. In this state he entered the Hôpital Cochin, which he soon left, without any notable improvement. He subsequently entered several other hospitals, but continued in the same state, complaining constantly of the cephalalgia and of the feebleness. Finally, about the commencement of October, he had an attack of epilepsy, which was renewed several times, and in which the convulsive movements occurred over the whole right side of the body. After the first attack he entered our service.

On his entrance his answers were vague, incoherent, and unsatisfactory; his physiognomy stupid; his eyes haggard; there was evidently a good deal of derangement in the intellect; the cephalalgia and the feebleness of the left arm and leg continued to progress. At this epoch both pupils were equally dilated.

On the 1st November he had another epileptic fit, during which convulsions occurred only in his left arm. This attack was followed by an attack of somnolency, which lasted some hours. From this time the feebleness rapidly increased, and his sight, which hitherto had been good, now became so much impaired that he could hardly distinguish objects.

In this condition the patient remained, without any apparent change, for nearly two months, when, at the visit a few days since, we found him in a state of stupor, from which nothing could rouse him. We found, on inquiry, that several times previously he had been similarly attacked, but that after a short time he recovered. Believing, consequently, that he was only suffering from an habitual attack, from which he would soon recover, we did not pay any particular attention to him. But the day following we found him in the same state; he was motionless, and insensible to all external excitement; his eyes were open, but totally insensible to light. His left arm, on being raised, fell like an inert mass. In the right arm some degree of muscular contractility seemed to remain. All the usual means, as sinapisms, &c., had been tried without result. There were here evident symptoms of an acute affection being added to the chronic one already existing; either an inflammation of the substance of the brain developed around some primitive lesion, or a serous effusion producing compression. The primitive affection was either a tumor or softening, or a chronic phlegmasia of the membranes. At the expiration of the fourth day the patient fell into complete coma and died.

On examination, after death, we found an encysted tumor of the size of a hen's egg, with thin walls, and filled with an opaque serous fluid, occupying the anterior part of the left cerebral hemisphere, but so near the mesian line, that it pushed somewhat the septem lucidum to the opposite side. This tumor was divided into two parts; one, the most voluminous, situated anteriorly; the second, smaller, posteriorly. Between these tumors there was a kind of strangulation, formed by a bridle, which separated completely the two tumors. In the smaller one we found an hydatid, in a collection of serum. The cerebral mass was softened to a considerable extent around the cyst. The seat of this tumor so near the optic thalami, and the origin of the motor nerves of the eye, explains the loss of vision; but the hemiplegia is not so easily accounted for, without it was due to the softening of the cerebral mass.

*Paris, February*, 1843.

